# Metabolomics based plasma biomarkers for diagnosis of oral squamous cell carcinoma and oral erosive lichen planus

**DOI:** 10.7150/jca.59777

**Published:** 2022-01-01

**Authors:** Xibo Li, Liwei Liu, Na Li, Qingquan Jia, Xiaoshuang Wang, Lihua Zuo, Jianglan Long, Peng Xue, Zhi Sun, Hongyu Zhao

**Affiliations:** 1 Department of Oral Emergency, The First Affiliated Hospital of Zhengzhou University· Stomatological Hospital of Henan Province, Zhengzhou, Henan, 450052, China.; 2 School and Hospital of Stomatology of Zhengzhou University, Zhengzhou, Henan, 450052, China.; 3 Department of Pharmacy, the First Affiliated Hospital of Zhengzhou University, Zhengzhou, Henan, 450052, China.; 4 Henan Engineering Research Center of Clinical Mass Spectrometry for Precision Medicine, Zhengzhou, Henan, 450052, China.; 5 Department of Prosthodontics, The First Affiliated Hospital of Zhengzhou University· Stomatological Hospital of Henan Province, Zhengzhou, Henan, 450052, China.; 6 Beijing Key Laboratory and Joint Laboratory for International Cooperation of Bio-characteristic Profiling for Evaluation of Rational Drug Use, Capital Medical University Affiliated Beijing Shijitan Hospital, Beijing, 100038, China.; 7 Health Management Center, The First Affiliated Hospital of Zhengzhou University· Stomatological Hospital of Henan Province, Zhengzhou, Henan, 450052, China.

**Keywords:** Oral squamous cell carcinoma, Oral lichen planus, Metabolomics, Biomarker, Plasma

## Abstract

**Backgrounds:** To identify diagnostic biomarkers for differentiating oral squamous cell carcinoma (OSCC) from oral erosive lichen planus (OELP) and investigate potential biomarkers associated with malignant transformation.

**Methods:** In this study, 72 patients with OSCC, 75 patients with OELP subjects were recruited. Their plasma samples were analyzed by ultra-high-performance liquid chromatography quadrupole-Orbitrap high-resolution accurate mass spectrometry, (UHPLC/Q-Orbitrap HRMS). Principal component analysis, orthogonal partial least square discrimination analysis, *t*-test analysis and false discovery rate were used to identify different metabolites in patients with OSCC and OELP. The metabolic pathway analysis was performed by MetaboAnalyst. To further screen and identify the biomarkers of OSCC and establish a diagnostic panel, binary logistic regression analysis and receiver operating characteristic analysis were used. The data were then combined with blood samples from healthy individuals for mass spectrometry analysis to obtain biomarkers related to malignant transformation.

**Results:** A total of 20 kinds of endogenous metabolites were identified from plasma samples of OSCC patients and OELP patients. Metabolic pathway analysis showed that the biomarkers associated with OSCC were closely related to cholic acid metabolism and amino acid metabolism. Finally, a diagnostic panel composed of decanoylcarnitine, cysteine and cholic acid was established. This diagnostic panel had good diagnostic efficiency with the AUC=0.998. Other metabolites including uridine, taurine, glutamate, citric acid and LysoPC(18:1) were identified to be general biomarkers for malignant transformation of OELP.

**Conclusion:** Biomarkers based on plasma metabolomics are of great significance for the prediction of malignant transformation of OELP and early diagnosis of OSCC.

## Introduction

Oral squamous cell carcinoma (OSCC) is one of the most prevalent tumors of head and neck and its 5-year survival rate is about 50%-60% 1. According to the Globocan Project (http://globocan.iarc.fr/Default.aspx) data, there are about 300 000 new cases every year, and 145 000 of them will die 2. A major cause for the high mortality of OSCC is that there is a lack of effective biomarker for early-stage diagnosis. The high mortality rate of OSCC is largely attributed to the fact that early-stage OSCC is mostly asymptomatic. In addition, the maxillofacial region has abundant blood and lymphatic vessels that facilitate invasion and metastasis of cancer cells. Therefore, most of the patients have advanced stage disease at diagnosis. Oral lichen planus (OLP) is the precancerous change of OSCC. Oral erosive lichen planus (OELP), a subtype of oral lichen planus, has a higher malignant transformation rate than the reticular type. The clinical presentations, especially oral mucosa erosions, are similar to the presentations of OSCC 3. It is difficult to distinguish early OSCC from OELP. There is an urgent need to distinguish OELP from OSCC to better the diagnosis and treatment.

Metabolomics is one of the typical hallmarks of cancer cells and is closely related to cancer development. Cancer cells maintain rapid proliferation by reprogramming their metabolic mechanisms 4. In general, metabolomics leads to abnormal levels of differential metabolites in blood, saliva and tissues. The changed metabolites may be potential biomarkers to distinguish malignant and benign lesions. Metabolomics is a high-throughput technique which is to measure the expression levels of small molecular compounds 5, such as lipids 6, amino acids 7, and other small molecule compounds, in body fluids or tissues 8. Theoretically, detecting metabolic change is a feasible method for diagnosis of OSCC.

Recently, many metabolomic studies have been performed to detect metabolic changes in patients with OSCC and healthy controls. Some study groups 9, 10 successfully revealed metabolites as potential biomarkers and used these biomarkers to discriminate OSCC patients from healthy control. Salivary biomarkers have attracted attention for clinical diagnosis because of the noninvasive sampling method 11, 12. Nevertheless, there is few studies 13 focused on searching for reliable plasma biomarkers of OSCC and OELP. Blood is more stable and contains more analytes compared with saliva 14. Several typical tumor specific proteins in blood, such as alpha fetoprotein (AFP) 15, prostate specific antigen (PSA) 16 and carcinoembryonic antigen (CEA) 17, have been used as biomarkers for clinical diagnosis. Our team has done some studies about diagnostic biomarkers on OELP and obtained some promising results 18. On the basis of previous research, the study aims to identify diagnostic biomarkers from metabolic reprogramming combined with mathematical model analysis, and build a reliable diagnostic panel. We also explored whether there were biomarkers that could predict the malignant transformation of OELP. The study will provide new ideas for the early diagnosis and treatment of OSCC, which has economic value and social significance.

## Materials and methods

### Sample Information

The study protocol was approved by the Ethics Committee of The First Affiliated Hospital of Zhengzhou University (approval No. 2020-KY-036). Written informed consent was obtained from all participants. All patients were diagnosed clinically and confirmed by pathology. Their blood samples were collected between June 6, 2019 and January 18, 2020. All the subjects were diagnosed as OSCC or OELP for the first time with no serious systemic disease. The OELP criteria for the diagnosis of OELP are the modification WHO diagnostic criteria of oral lichen planus 19: In short, clinical criteria included presence of bilateral, more or less symmetrical lesions accompanied by erosion. Moreover, the histological criteria included a clearly defined band-like zone of cellular infiltration which is limited to the surface part of connective tissue, mainly composed of lymphocytes, in the basal cell layer, signs of 'liquefactive degeneration' and absence of epithelial dysplasia. Clinical examinations were performed by two chief physicians with more than 30-year clinical experience. Also, every patient with OELP had undergone histopathological examination. In total, 72 patients with OSCC, 75 patients with OELP were recruited in this study. To detect a 50% difference in peak areas of each ion peak with a sample size of 100 in total, we could obtain a power over 0.99. In our case, the differences in peak areas between OSCC and OELP groups are mostly more than 50%. Therefore, a total number of 147 patients is a reasonable sample size to detect the difference. The original dataset was divided randomly into training (n=98) and validation (n=49) sets in a ratio of 2:1, according to random number method. Samples were collected and stored at -80°C before UHPLC-Q-Orbitrap analysis.

### Experimental equipment

Thermo Fisher centrifuge (Thermo Fisher Science, USA); VORTEX-GENIE 2 vortex oscillator (SITM, USA); BX7200HP ultrasonic cleaner (Shanghai Xinmiao medical device manufacturing Co., Ltd.); AL104 balance with accuracy of 0.0001 (Mettler Toledo Shanghai Co., Ltd., Switzerland); CF16RN centrifuge (Hitachi, Japan); Ultrapure water meter (MilliPore, USA); and -80 °C ultra-low temperature refrigerator (Thermo Fisher science, USA).

### Experimental reagent

Acetonitrile (Chromatographic grade, Fisher, USA); Methanol (Chromatographic grade, Fisher, USA); Formic acid (Chromatographic grade, Shanghai Aladdin Biotechnology Co., Ltd., China); L-2-chlorophenylalanine (Bailingwei Technology Co., Ltd., China); Ketoprofen (Sigma, USA); all solutions were filtered by 0.22 μm aperture filter before used.

### Sample collection

All blood samples were collected from 8:00 a.m. to 10:00 a.m. The blood was collected to the vacuum tubes containing coagulant, placed in the incubator containing ice. The sample was centrifuged at 1510×g for 10 minutes at 4°C, and the supernatant was quickly stored at -80°C until used.

### Sample preparation

100 μL sample was taken out and placed in a 1.5 mL centrifuge tube after thawing. 300 μL methanol solution containing internal standards (0.05 μg/mL L-2-chlorophenylalanine and 0.5 μg/mL ketoprofen) was added. After vortex oscillation for 1 min, centrifugation was performed at 16 200 × g for 10 min at 4°C. The supernatant was aspirated to the vial for analysis.

### QC sample preparation

Quality control sample (QC sample) analysis could ensure the reliability of the experimental results in the process of collecting metabolomics data of all samples. 6 QC samples were detected to monitor the pressure change before and after each injection and the shift of the main peak retention time of the total ion flow diagram. After the instrument was stable, the sample analysis started. A QC sample was inserted into every ten samples to verify the stability of the instrument. Inserted a blank sample containing only solvent after each QC sample to avoid cross-contamination.

### UHPLC-Q-Orbitrap analysis

UHPLC-Q-Orbitrap system: Ultimate 3000 UHPLC (Thermo Fisher Scientific, USA), Q Exactive high resolution mass spectrometry (Thermo Fisher Scientific, USA); ACQUITY UHPLC^®^RBEH C_18_ (100 × 2.1 mm, 1.7 µm) column (Waters Company, USA).

Metabolomic analyses of plasma samples were as described previously 20. The ultra-performance high liquid chromatography (UHPLC) system was used to separate the metabolites in plasma samples. 5 µL was extracted from each sample and injected into the ACQUITY UHPLC^®^RBEH C_18_ column (100 × 2.1 mm, 1.7 µm), and the column temperature was 40°C. The mobile phase was acetonitrile (A) with 0.1% formic acid aqueous solution (B), and gradient elution flow rate was 0.2 mL/min:0.0~1.0 min, 5%A; 1.0~9.0 min, 5%~100%A; 9.0~12.0 min, 100%A; 12.0~12.1 min, 100%~5%A; 12.1~15.0 min, 5%A.

Heated electrospray ionization (HESI) was combined with high resolution mass spectrometry to UHPLC system. The temperature of auxiliary gas was 300 °C and the flow rate was 10 arb. The ion source and capillary were 350 °C and 320 °C. The detection was performed in positive ion mode and negative ion mode with a resolution of 17 500 in full mass/DDMS^2^ (data dependent mass spectrometry) scanning mode. The collision energy was set at gradient from 20 eV to 60 eV. The spray voltage and sheath gas flow rate were 3.50 kV and 40 arb in positive ion mode, and 2.80 kV and 38 arb in negative ion mode. The injection sequence of all samples was random.

### Data processing and statistical analysis

Data were tested for normality using a Shapiro-Wilk normality test. When the normal distribution was satisfied, an independent-samples *t*-test was applied. Otherwise, a non-parametric Wilcoxon test was performed. All metabolomics data were analyzed by Thermo Xcalibur™ software (Version 3.0, Thermo Scientific, USA). Specific parameters were as previously 18. Finally, the generated data and the m /z value, retention time (RT) and peak area of each ion peak in each sample were collected. The peak areas represented the levels of metabolites. The data sets were imported into the multivariate statistical analysis software SIMCA (Version 14.0, Umetrics, Sweden). The principal component analysis (PCA) and orthogonal partial least square discriminant analysis (OPLS-DA) were performed to explore separation trend among groups. Through the establishment of the OPLS-DA model, variable importance in projection (VIP value) was obtained. Two hundred permutation tests used to evaluate whether the data was overfitted. *P* values were obtained using the independent-samples *t*-test. Also, in order to further screen the metabolites with significant difference between different groups, the false discovery rate (FDR) which was calculated by R language was conducted for metabolites with VIP value greater than 1 using SPSS 26.0 software (IBM, USA). Eventually, the metabolites were selected for identification when FDR < 0.05. The accurate m / z, ion chromatogram, retention time (RT) and other information were compared with ChemSpider and MassList database. The MS/MS data was compared with mzVault, Human Metabolome Database (HMDB, http://hmdb.ca/) and PubChem compound database. For some endogenous metabolites of which the standard substance could be obtained, the data were compared with the standard substance to determine its structure. When the data matched with the information in database, the metabolite was considered to be identified successfully. Moreover, to screen the metabolites of OELP malignant transformation, fold changes and FDR of the potential biomarkers for comparisons of OELP versus HC and OSCC versus HC were calculated. Using MetaboAnalyst (www.metaboanalyst.ca) platform, the screened differential metabolites were analyzed by thermography to show the change of the metabolites, and receiver operating characteristic (ROC) curve was drawn for each identified differential metabolite. Area under the curve (AUC) was calculated by Medcalc. A metabolic pathway network was formed according to Kyoto Encyclopedia of Genes and Genomes (KEGG, https://www.kegg.jp/kegg/pathway.html) signaling pathway database.

## Results

### Demographic baseline characteristics

**Figure 1:**
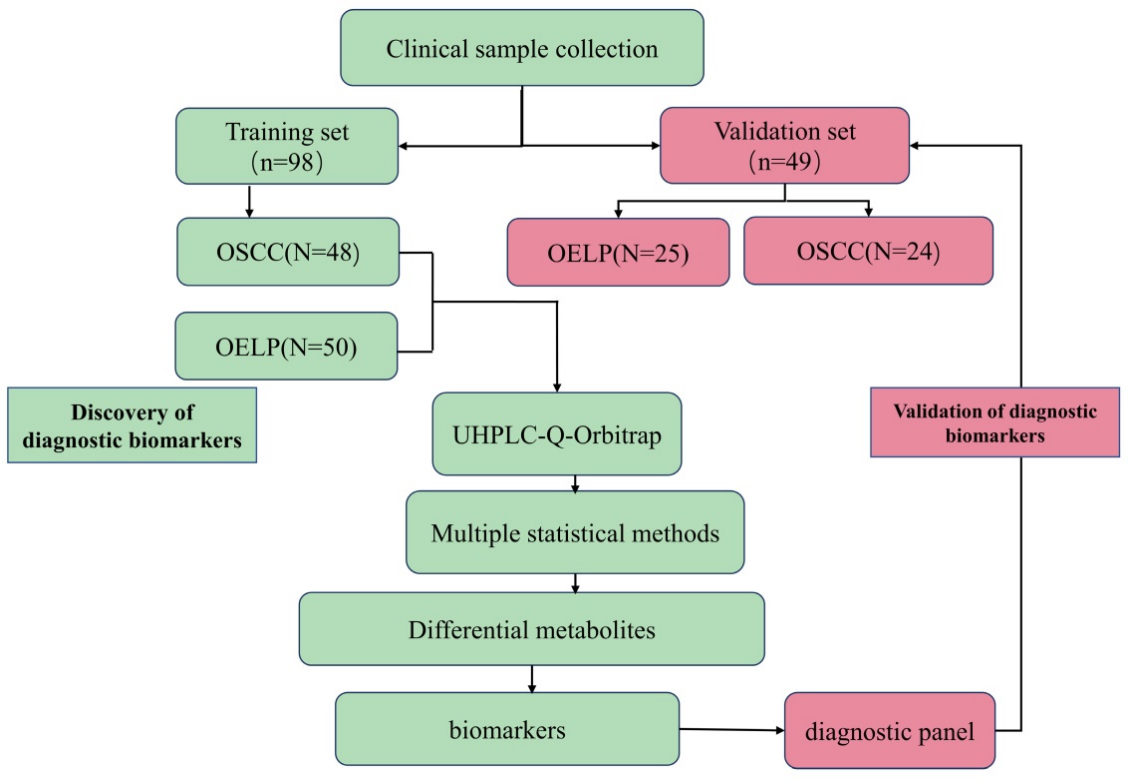
Flowchart. OSCC: oral squamous cell carcinoma; OELP: oral erosive lichen planus; UHPLC/Q-Orbitrap HRMS: ultra-high-performance liquid chromatography quadrupole-Orbitrap high-resolution accurate mass spectrometry.

The flowchart of the study was shown in Figure 1. A total of 147 subjects were enrolled in training group including 72 patients with OSCC with a mean age of 66 ± 12 (yrs.; mean ± SD), 75 patients with OELP with a mean age of 61 ± 7 (yrs.; mean ± SD). There were no significant differences in gender, age, BMI and lifestyle habits of participants among these three groups. The data of 48 OSCC patients and 50 OELP patients were used for biomarker discovery and the others for validating the effectiveness of the chosen biomarkers. The demographic baseline characteristics of these individuals were shown in Table 1.

### Primary metabolites analysis in blood samples

**Figure 2:**
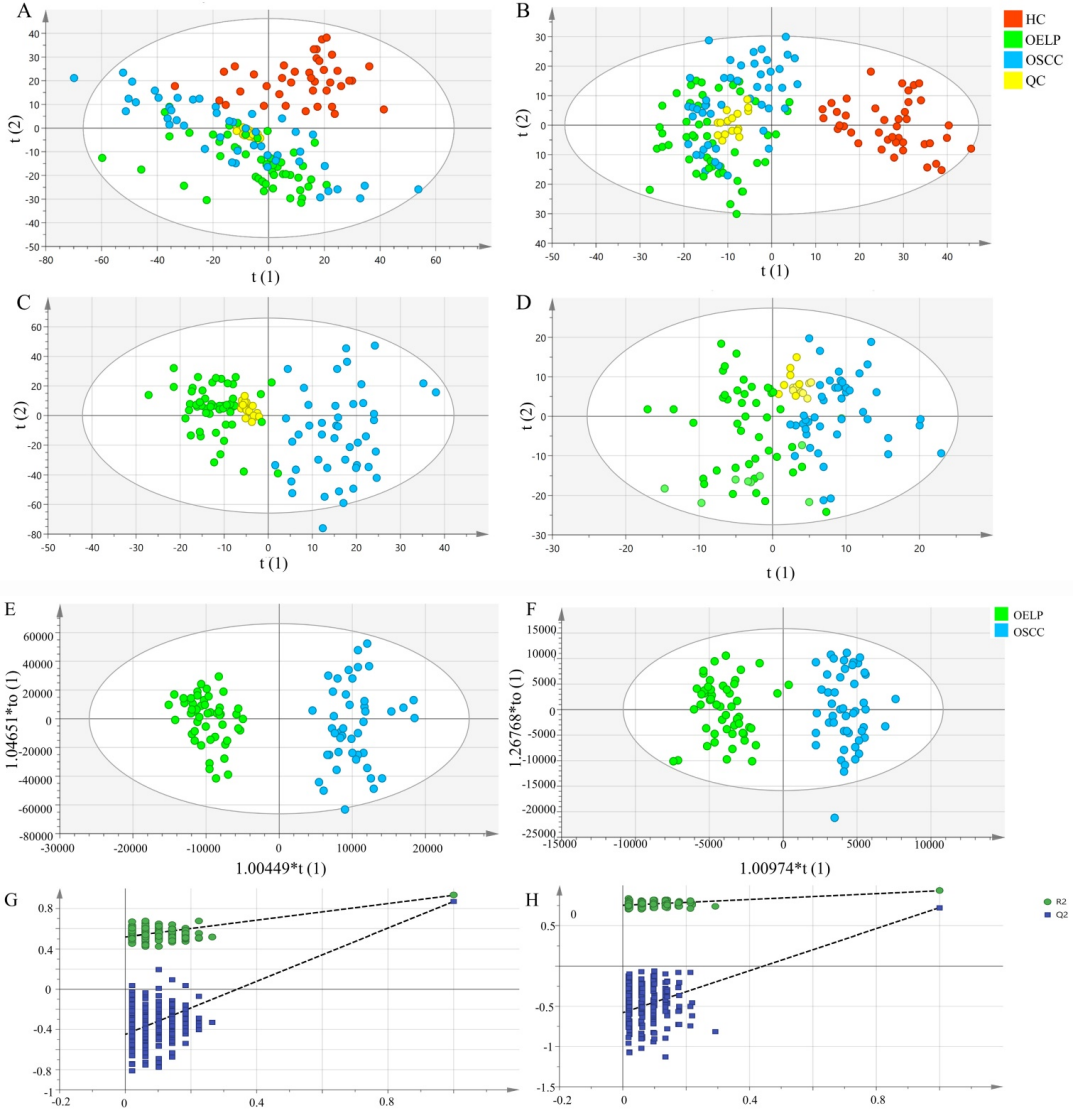
Multivariate statistical analysis revealed distinct metabolic characteristics between OSCC and OELP. HC: red pots; OELP: green pots; OSCC: blue pots; QC: yellow pots. PCA score plots among OSCC, OELP and HC in (A) positive ion mode and (B) negative ion mode built with metabolites identified by LC-MS. The abscissa and the ordinate represented the first and second principal component. PCA score plots of the OSCC and OELP group in (C) positive ion mode and (D) negative ion mode built with metabolites identified by LC-MS. The abscissa represented and the ordinate represented the first and second principal component. OPLS-DA score plots of the OSCC and OELP in (E) positive ion mode and (F) negative ion mode. The model showed a clear trend of separation between OSCC and OELP. Cross-validation plots with a permutation test repeated 200 times of the OSCC and OELP groups in (G) positive ion and (H) negative ion mode. It showed that the OPLS-DA models were not overfitting. PCA: principal component analysis; OPLS-DA: orthogonal partial least square discriminant analysis; HC: healthy control; OSCC: oral squamous cell carcinoma; OELP: oral erosive lichen planus.

To gain insights into the metabolic features of OELP progressed to OSCC, UHPLC/Q-Orbitrap HRMS was performed on blood samples of 48OSCC, 50 OELP and 47 healthy controls (HC). A total of 3238 ion peaks in positive ion modes and 2663 in negative ion modes were extracted. The metabolic dataset was next analyzed by PCA. QC samples cluster tightly together indicating the good instrument stability and the reliability of the data. As shown in Figure 2A-B, the disease groups, including both the OSCC group and the OELP group, were clearly separated from HC, but there was not a sharp distinction between OSCC and OELP group. This result confirmed that it is difficult to distinguish OELP from early OSCC in clinical diagnosis, but the difference between healthy people and disease groups is very clear. Therefore, we further analyzed differential metabolites between OSCC and OELP group.

### Screening and identifying differential metabolites

In order to further explore the unique metabolic characteristics between OSCC and OELP, PCA analysis were carried out. There was a clear trend of inter-group separation between the two groups. All samples were analyzed in both positive (Figure 2C) and negative (Figure 2D) ion modes. To better define the metabolic variations in OSCC and OELP, OPLS-DA analysis were performed. As shown in Fig. 2E-F, green dots represented patients of OELP and blue dots represented patients of OSCC. R^2^Y = 0.932, Q^2^ (cum) = 0.870 in positive ion mode and R^2^Y=0.905, Q^2^ (cum) = 0.698 in negative ion mode. The results indicated significant separation between groups. Two hundred permutation tests were further performed, with R^2^ = (0.0, 0.519), Q^2^= (0.0, -0.450) in positive ion mode and R^2^ = (0.0, 0.666), Q^2^= (0.0, -0.503) in negative ion mode, which confirmed that these OPLS-DA models were not overfitting (Figure 2G-H).

**Figure 3:**
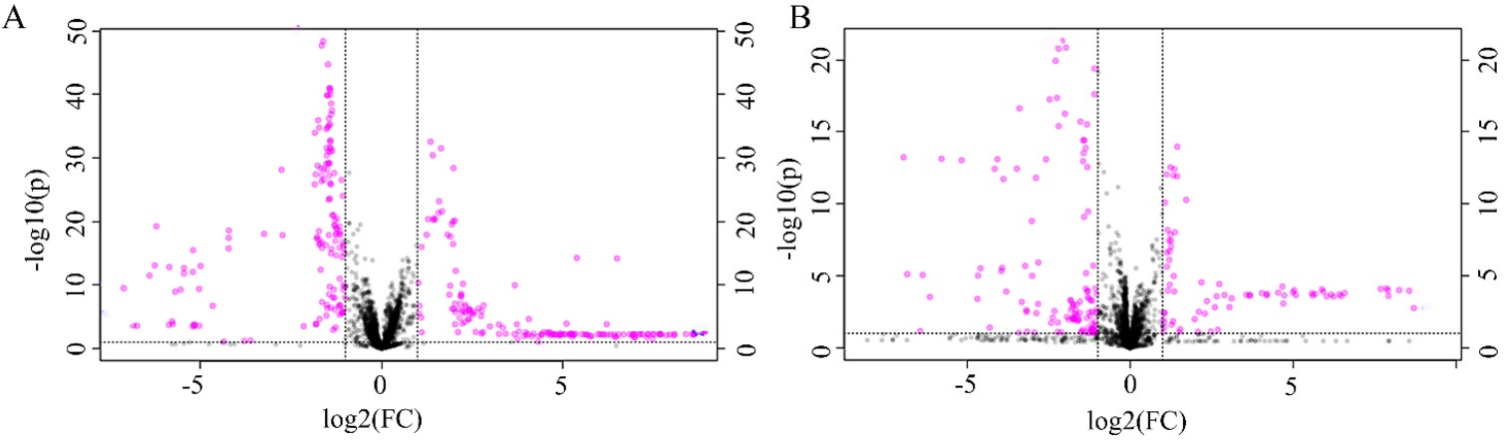
The volcano plot of the OSCC vs. OELP in (A) positive ion mode and (B) negative ion mode. OSCC, oral squamous cell carcinoma; OELP, oral erosive lichen planus.

**Figure 4:**
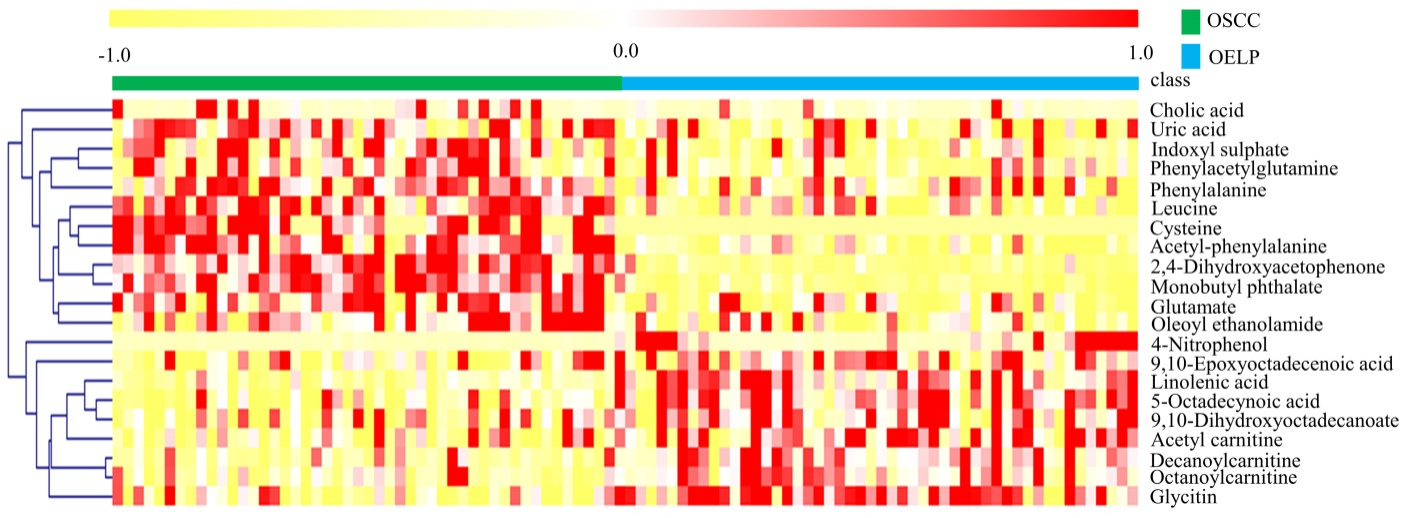
Heat map of 20 differential metabolites showing significant differences between the oral squamous cell carcinoma and oral erosive lichen planus (OELP) groups. Red and yellow represented higher and lower concentrations. The abscissa represented the number of subjects. The ordinate represented differential metabolites of the two groups. Pearson's correlation-based clustering analysis was used for clustering analysis of 20 differential metabolites. OSCC, oral squamous cell carcinoma; OELP, oral erosive lichen planus.

**Figure 5:**
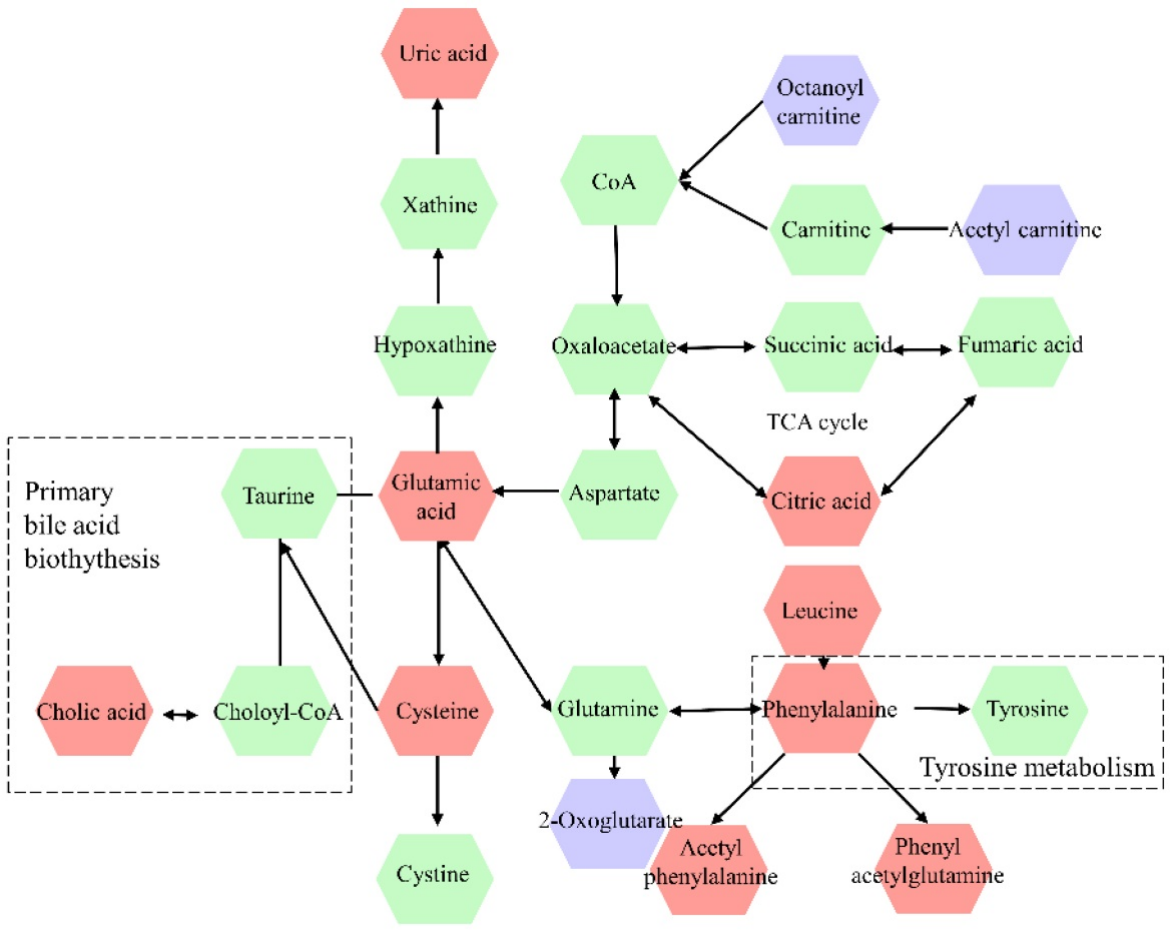
Metabolic pathway network diagram of differential metabolites in OSCC and OELP. The violet color represents downregulated metabolites, red color represents upregulated metabolites, the green color metabolites represent intermediary metabolites in the metabolic pathway of OSCC. OSCC, oral squamous cell carcinoma; OELP, oral erosive lichen planus.

The differential metabolites were further screened by combining the *P* values or the fold change and VIP values of the OPLS-DA model. Volcano plots were drawn using fold change (FC) and *P* values. Red dots represented metabolites with* P* < 0.05 (- log10*P* > 1.30) and FC > 2.0 (log2FC > 1.0). The sites with VIP > 1 and *P* < 0.05 were regarded as candidate differential metabolites (Figure 3). After comparison with databases, a total of 20 endogenous metabolites between OSCC and OELP were identified. These endogenous metabolites included amino acids such as cysteine, glutamate, phenylalanine; lipids, such as lysophosphatidylcholine (LysoPC) and other small-molecule compounds. The details of these metabolites were given in Table 2. The heatmap of the differential metabolites were shown in Figure 4 which appeared the changes in metabolic signatures among OSCC and OELP. In order to better understand the relationship among metabolites, the metabolic pathway network diagram was shown in Figure 5.

### Pathway analysis

**Figure 6:**
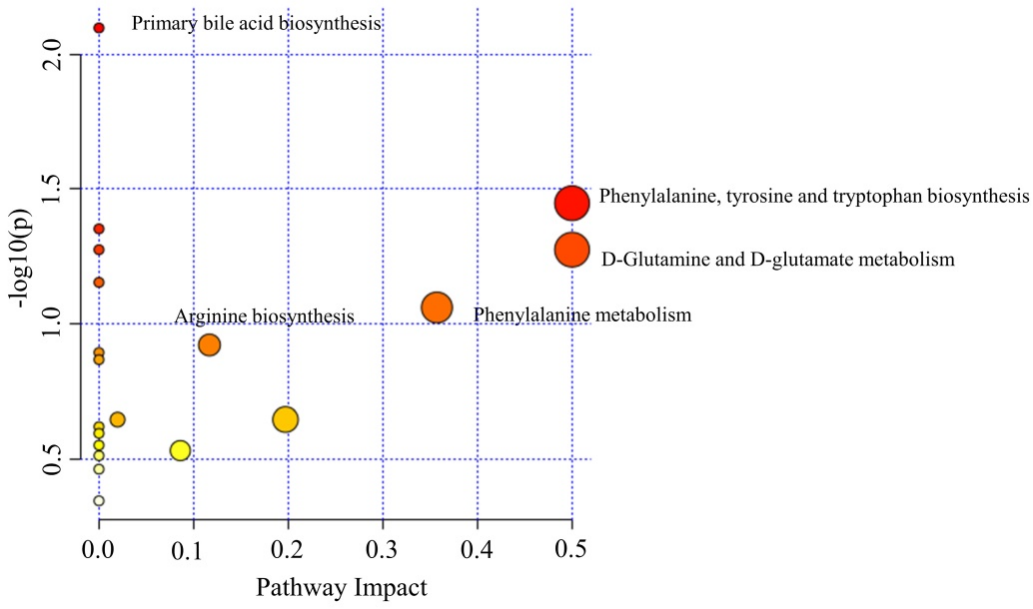
The disturbed metabolic pathways showed various metabolic changes in OSCC and OELP group. OSCC, oral squamous cell carcinoma; OELP, oral erosive lichen planus.

To further explore the underlying molecular mechanism of OSCC, the metabolic pathways of the metabolites were analyzed by MetaboAnalyst (Figure 6). The results showed that amino acid metabolism, including phenylalanine, tyrosine and tryptophan biosynthesis, D-Glutamine and D-glutamate metabolism, phenylalanine metabolism; primary bile acid biosynthesis and arginine biosynthesis were associated with OSCC. Phenylalanine, tyrosine and tryptophan biosynthesis and D-Glutamine and D-glutamate metabolism had a major impact on OSCC.

### Establishment of a diagnostic panel

**Figure 7:**
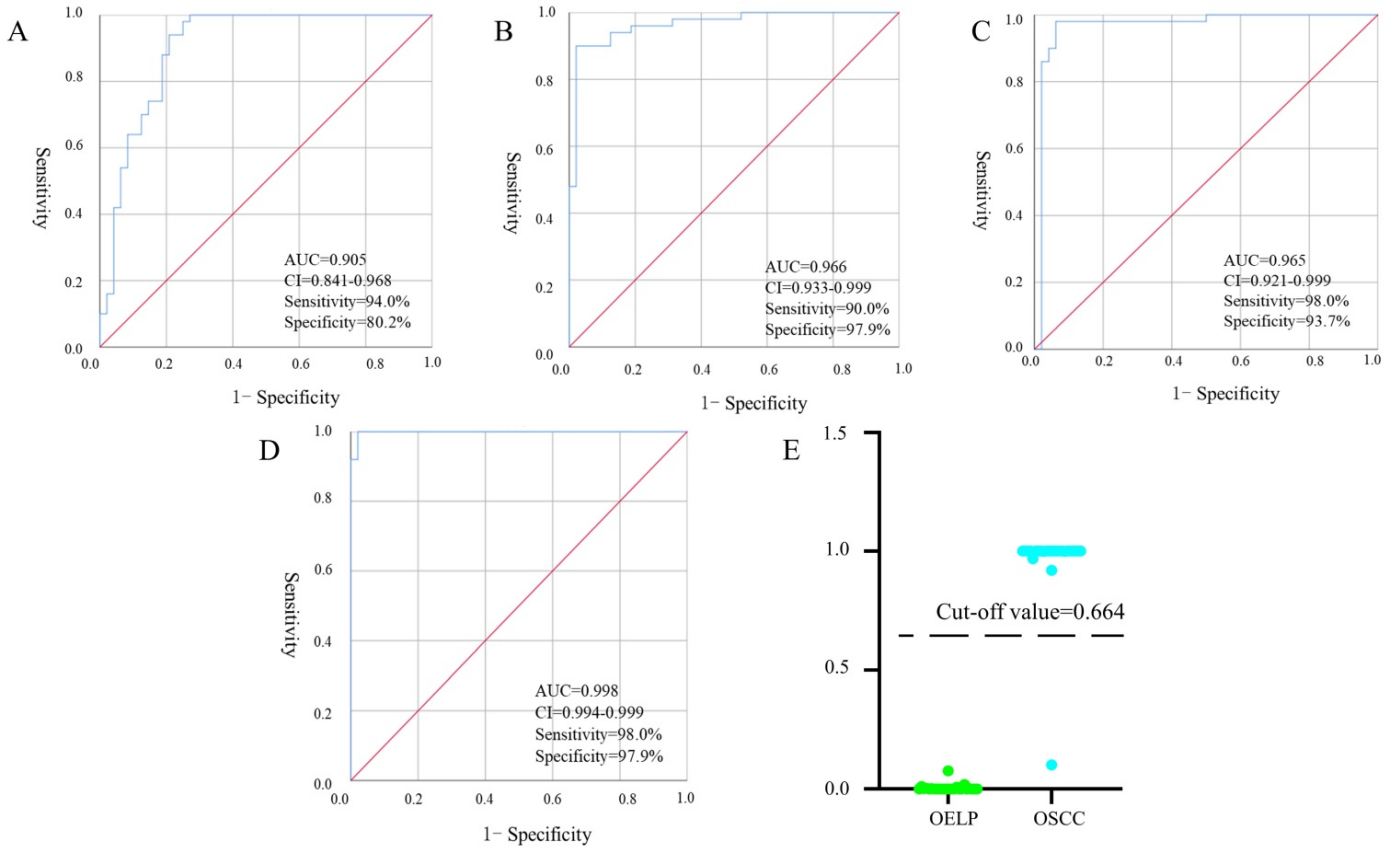
Receiver operating characteristic curves of multiple logistic regression models with (A) decanoylcarnitine, (B) cysteine, (C) cholic acid and (D) biomarker panel. (E) The prediction accuracy of the diagnostic panel in validation set. OSCC, oral squamous cell carcinoma; OELP, oral erosive lichen planus; QC, quality control sample; AUC, area under curves; 95%CI, 95% confidence interval.

Binary logistic, ROC analysis and VIP were used to evaluate integrated biomarkers. ROC curve was used to assess the diagnostic performance of each metabolite. Subsequently, the metabolites of AUC > 0.900 were used to establish a diagnostic panel. The panel with reasonably high accuracy and sensitivity exhibited well-established performance. All 95% confidence intervals were given in the form: (95%CI lower, upper). Ultimately, decanoylcarnitine (Figure 7A), cysteine (Figure 7B) and cholic acid (Figure 7C) were selected to serve as a useful biomarker panel for the diagnosis of OSCC from OELP. The FC of decanoylcarnitine, cysteine and cholic acid in the observation group were 0.465, 54.585, 3.509, respectively (Table 2), the difference was statistically significant (*P* < 0.05). ROC analysis showed that the diagnostic capability of biomarker panel (95%CI 0.904, 0.999, *P* < 0.0001) (Figure 7D) was much higher than those of previous biomarkers of OSCC, including decanoylcarnitine (95%CI 0.841, 0.968, *P* < 0.0001), cysteine (95%CI 0.933, 0.999, *P* < 0.0001), and cholic acid (95%CI 0.921, 0.999, *P* < 0.0001).

According to the ROC curve, the Youden's index was calculated. The best cut-off value was 0.664. It was used to distinguish OSCC from OELP in the verification set. Samples above the cutoff value were diagnosed as patients with OSCC and below the cutoff value were diagnosed as patients with OLP. Only one case was wrong when the diagnostic panel was used to diagnose the validation set. The results presented that the panel achieved a diagnostic accuracy of 97.9% (Figure 7E).

### Discovery of malignant transformation biomarkers

**Figure 8:**
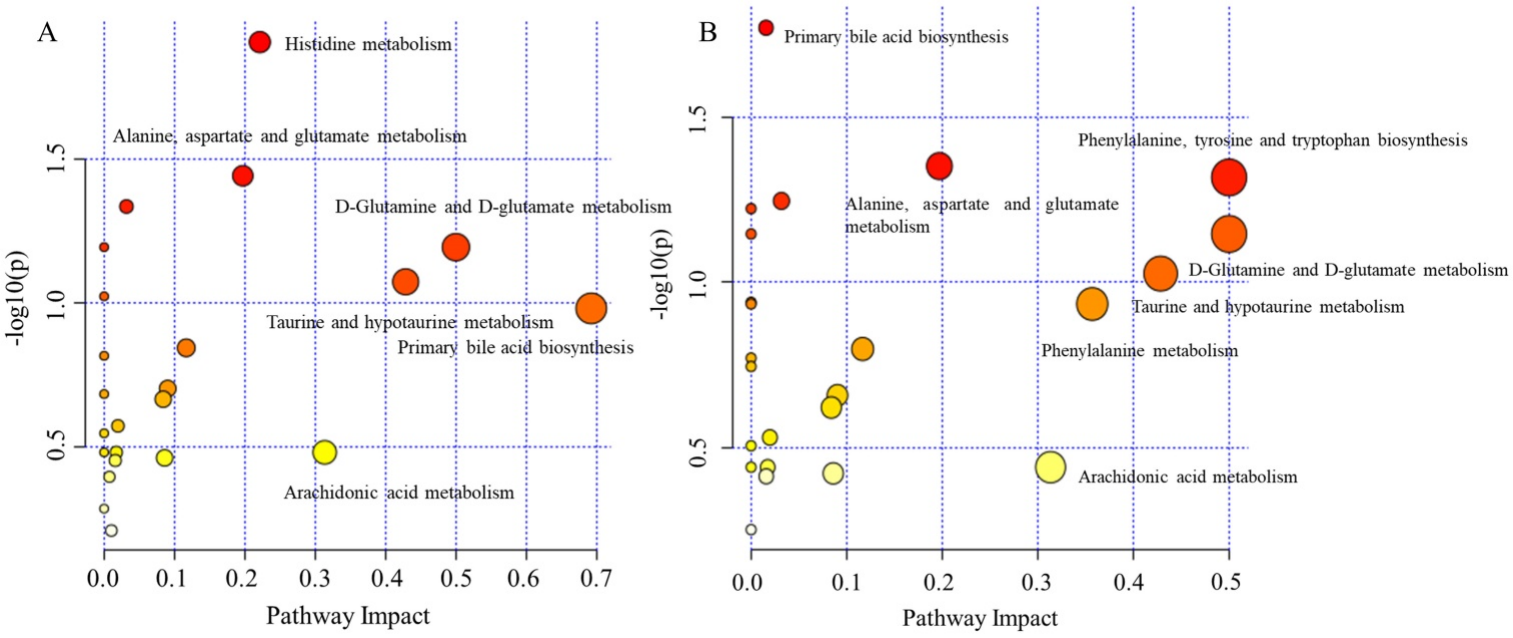
The disturbed metabolic pathways showed various metabolic changes in (A) OELP and HC group and (B) OSCC and HC group. OSCC, oral squamous cell carcinoma; OELP, oral erosive lichen planus; HC, healthy control.

In order to gain additional insight into the metabolic features when OELP progressed to OSCC, plasma metabolomic analysis was performed to distinguish metabolites in OSCC, OELP and healthy controls. An additional group of 47 healthy volunteers were recruited as healthy controls (HC), who were well matched with aspects of age, gender, and body mass index (BMI) of the patients. 47 healthy controls with a mean age of 65 ± 9 (yrs.; mean ± SD). The demographic baseline characteristics were shown in Table 1. The metabolites of OSCC and HC were listed in Table 3 and OELP and HC were listed in Table 4. Both OSCC and OELP patient groups showed decreased FC in uridine, taurine, glutamate, citric acid and LysoPC (18:1). There was a significant difference (FDR < 0.05) between disease group and HC. However, the OSCC group showed a more pronounced decreased compared to OELP. Altered metabolites could possibly contribute to cancer progression. The metabolic pathway analysis was performed respectively and some common disturbed metabolic pathways were found. D-Glutamine and D-glutamate metabolism, primary bile acid biosynthesis, alanine, aspartate and glutamate metabolism, taurine and hypotaurine metabolism and arachidonic acid metabolism may be associated with the malignant change of OELP (Figure 8).

## Discussion

Despite the continuous advancement of diagnostic and treatment modalities in the past 20 years, the 5-year survival rate of OSCC has not substantially improved 21. Besides, there are no specific symptoms in the early stage of the disease, and it is difficult to make a diagnosis by pathological biopsy in the early stage. These could result in a series of consequences such as pain, bleeding, infection and even death. Thus, our research group has been seeking a diagnostic method which is accurate, convenient, and less harmful. We focused on blood samples because the blood contained metabolites that could be used for successful identification of diagnostic biomarkers. The imbalance of metabolites might be associated with pathological mechanism of OSCC.

One of the central results of our study was that some biomarkers decreased more in OSCC compared to OELP. Previous studies only showed that biomarkers in OSCC or OELP by comparing healthy controls, but few studies have focused on the metabolic alterations precede malignant transformation of OELP into OSCC. In our study, we found that the level of uridine, taurine, glutamate, citric acid and LysoPC (18:1) were lower in OELP than in OSCC. Uridine was generally thought safe and harmless, but recent studies found that uridine homeostatic disorder was carcinogenic. Uridine could be used to synthesize deoxyuridine triphosphate (dUTP). While dUTP was likely to cause errors in DNA replication. DNA damage may cause or greatly increase a person's susceptibility to cancer 22, 23. It also made a person who had a higher likelihood of OSCC. In addition, uridine synthesis originated from glutamine and glutamine could be synthesized from glutamate and ammonia 24. Glutamate was regarded as a potential diagnostic biomarker of OSCC in a previous study 25. Amino acids are an important unit of energy source for basic metabolic pathways in human beings. The abnormalities in amino acid metabolism may be a unique sign of OSCC 26. There was no doubt that proliferation of OSCC cells required more energy consumed than OELP. Therefore, compensatory mechanisms promoted excessive consumption of amino acids to maintain the normal physiological function in the body, which could further aggravate metabolic disorders and exacerbate the disease. Taurine could cause tumor cell apoptosis 27, showing anti-cancer effects 28. In our study, taurine was greatly reduced in OSCC patients, which may be related to the proliferation of OSCC cells. The proliferation of OSCC cells may in turn inhibit the anti-tumor effect of taurine. From HC to OELP to OSCC, the decrease of uridine, glutamate and taurine may be used for the mass synthesis of damaged-DNA. Cancer cells display diverse metabolic reprogramming including reductive carboxylation in the citric acid cycle to use glutamine into intracellular lipid storage 29. Hence, citric acid and LysoPC(18:1) may be considered as the biomarker for the transformation of OELP to OSCC because progression of OSCC could exhaust more energy. Collectively, our data indicated a microenvironment that was conducive to the rapid proliferation of cancer cells was gradually formed in OSCC patients.

Previous studies on metabolomics in OSCC included healthy individuals who were regarded as controls for OSCC group 30. Although it was a common method when identifying diagnostic biomarkers, it didn't consider potential malignant lesions which could alter metabolic features. Currently, approximately 28 000 000 of OELP occur in the world 31 and 1.1% of OLP may transform into a malignant cancer 32. Selecting OELP patients as a control group in OSCC studies could minimize confounding factors related to benign diseases.

Another important finding in this study was a biomarker panel of OSCC and OELP. As we know, any single diagnostic biomarker had limited diagnostic accuracy (Figure 7A-C). When we combined the four biomarkers together as a “diagnostic panel”, the panel offered superior diagnostic performance and showed significantly higher sensitivity (Figure 7D). Further understanding of these biomarkers may provide more insight into the pathogenesis of OSCC and serve new therapeutic interventions. A previous study showed that the level of acylcarnitine decreased in esophageal squamous cell carcinoma 33. Carnitine could affect metabolic mechanisms in numerous ways. Acyl-coenzyme A synthetases could catalyze the thioesterification of coenzyme A (CoA) to acyl-CoA esters while Acyl-CoA esters could be converted to acyl-carnitine esters by carnitine acyltransferases 34. Therefore, the decrease of decanoyl carnitine may result from the inhibition of the enzymes' activity and levels by OSCC cells. In addition, the incidence of lymph node metastasis and bone metastasis were associated with acyl carnitine 35. It was possible that decanoyl carnitine could favor the identification and demarcation of OELP and OSCC. Amino acid was an important source of energy storage, and its abnormal metabolism may be an important characterization of cancer 26. Cysteine had been regarded as a biomarker of oral cancer in both blood and saliva 36. Also, cysteine was the precursor for the formation of glutathione. Glutathione could regulate the redox state and immune response in the system 37. The decrease of cysteine may be related to the inhibition of immune system and a disorder in fatty acid oxidation metabolism by OSCC. Cholic acid could activate the TGR5 receptor which can induce OSCC cell proliferation 38. This result was in line with the increase of cholic acid production in our study. The result suggested that a microenvironment which was suitable for carcinogenesis was gradually formed in the body of OSCC patients for promoting the tumor growth.

Taken together, we used UHPLC-Q-HRMS to analyze the differential metabolites in the plasma of patients with OSCC and OELP. We found that metabolic characterization and metabolic pathways in OSCC patients were significantly different from those in OELP. These changes in endogenous metabolites and abnormal metabolic pathways may be related to the pathogenesis of OSCC, which could be used for the diagnosis of OSCC. This study provided a basis for clinical molecular diagnosis and had important significance for clinical diagnosis and treatment of OSCC. In the future, more patients, including those with cancers, precancerous lesions and healthy controls, will be recruited to further verify the clinical applicability of the biomarkers described in this study.

## Conclusion

In this study, a panel of metabolites that consist of decanoylcarnitine, cysteine and cholic acid was identified for the diagnosis of OSCC. The metabolites uridine, taurine, glutamate, citric acid and LysoPC(18:1) were found to be potential biomarkers indicating malignant transformation of OELP. Biomarkers based on plasma metabolomics could be very helpful for diagnosis of OSCC.

## Figures and Tables

**Table 1:** Demographic baseline characteristics of patients with OSCC, OELP and healthy controls

Characteristics	OSCC patients (n=72)	OELP patients (n=75)	Healthy controls (n=47)
Age (yrs.; mean ± SD)	66 ± 12	61 ± 7	65 ± 9
**Gender**			
Male	35	38	23
Female	37	37	24
**BMI**	21.06±2.81	21.74±2.40	20.83±2.98
Habit of chewing betel nut	7/72	5/72	4/72
**Habit of smoking (≥1 cigarette / d for more than half a year)**			
Yes	32/72	31/75	18/47
**Habit of eating spicy food**			
Yes	43/72	42/75	29/47
**Habit of eating very hot food**			
Yes	15/72	18/75	14/47
**Clinical stage**			
I	17		
II	21		
III	19		
IV	14		
Unknown	1		

BMI: Body mass index; Habit of chewing betel nut: often chewing betel nut; Habit of eating spicy food: often intake spicy food; Habit of eating very hot food: often intake very high-temperature food; OSCC: oral squamous cell carcinoma; OELP: oral erosive lichen planus; SD: standard deviation; confirmed by a histopathological examination.

**Table 2:** Metabolites different between OSCC and OELP

ID	Name	Formula	Molecular	RT	VIP	*P*	FC	AUC	FDR
1	Uric acid	C_5_H_4_N_4_O_3_	168.02019	1.02	4.353	0.009	1.170	0.677	0.013
2	Glutamate	C_5_H_9_NO_4_	147.04588	1.03	1.86	1.48E-06	1.279	0.780	3.27E-03
3	Acetyl carnitine	C_9_H_17_NO_4_	203.12286	1.03	2.641	4.42E-05	0.715	0.738	1.89E-04
4	Leucine	C_6_H_13_NO_2_	132.08640	1.48	1.034	1.95E-07	1.438	0.810	2.35E-05
5	Phenylalanine	C_9_H_11_NO_2_	165.08591	1.95	3.949	3.83E-07	1.235	0.686	3.25E-06
6	Phenylacetylglutamine	C_13_H_16_N_2_O_4_	264.10388	3.97	2.304	0.003	1.910	0.711	0.017
7	2,4-Dihydroxyacetophenone	C_8_H_8_O_3_	152.03912	4.19	1.047	5.48E-18	1.393	0.766	6.72E-13
8	Cysteine	C_3_H_7_NO_2_S	121.01138	4.47	1.513	7.35E-14	54.585	0.905	9.48E-12
9	Acetyl phenylalanine	C_11_H_13_NO_3_	207.08211	4.62	1.003	6.34E-13	1.819	0.888	0.005
10	4-Nitrophenol	C_6_H_5_NO_3_	139.01865	5.28	1.940	3.78E-05	0.143	0.822	4.16E-11
11	Indoxyl sulphate	C_8_H_7_NO_4_S	213.00214	5.5	3.750	3.82E-05	1.610	0.700	0.013
12	Octanoylcarnitine	C_15_H_29_NO_4_	287.21664	5.51	1.694	2.33E-05	0.616	0.763	1.09E-04
13	Glycitin	C_22_H_22_O_10_	446.12964	6.08	1.042	6.21E-06	0.783	0.817	3.53E-05
14	Decanoylcarnitine	C_17_H_33_NO_4_	315.24832	6.26	2.509	3.98E-06	0.465	0.966	2.39E-05
15	Monobutyl phthalate	C_12_H_14_O_4_	222.08189	6.52	1.830	7.04E-20	1.975	0.834	0.006
16	Cholic acid	C_24_H_40_O_5_	408.28156	6.8	3.46	0.009	3.509	0.965	0.012
17	9,10-Dihydroxyoctadecanoate	C_18_H_36_O_4_	316.25436	8.08	1.102	0.002	0.821	0.685	7.40E-09
18	9,10-Epoxyoctadecenoic acid	C_18_H_32_O_3_	296.22800	9.22	4.275	0.004	0.708	0.680	0.003
19	Oleoyl ethanolamide	C_20_H_39_NO_2_	325.30487	9.57	1.019	2.16E-07	1.555	0.717	1.99E-06
20	5-Octadecynoic acid	C_18_H_32_O_2_	280.23303	10.17	9.646	7.30E-08	0.600	0.823	5.45E-04

OSCC, oral squamous cell carcinoma; OELP, oral erosive lichen planus; FC, fold change; RT, retention time; VIP, variable importance in projection; FC, fold change; AUC, area under curve; FDR, false discovery rate, calculation based on Benjamini and Hochberg.

**Table 3:** Metabolites different between OSCC and HC

ID	Name	Formula	Molecular	RT (min)	VIP	*P*	FC	AUC	FDR
1	Uridine	C_9_H_12_N_2_O_6_	244.06227	1.00	1.028	1.02E-10	0.718	0.882	8.53E-10
2	Chiro-inositol	C_6_H_12_O_6_	180.05553	1.01	1.283	0.027	1.093	0.742	0.049
3	Lactate	C_3_H_6_O_3_	90.02322	1.01	1.158	0.026	1.089	0.632	0.047
4	Taurine	C_2_H_7_NO_3_S	125.00639	1.01	1.906	3.35E-13	0.662	0.906	4.07E-12
5	Glutamate	C_5_H_9_NO_4_	147.04501	1.02	1.907	1.96E-13	0.650	0.912	2.52E-12
6	Uric acid	C_5_H_4_N_4_O_3_	168.02017	1.02	2.858	8.53E-10	0.882	0.620	0.021
7	Acetylcarnitine	C_9_H_17_NO_4_	204.12285	1.02	1.662	1.23E-05	0.682	0.774	2.9E-03
8	Citric acid	C_6_H_8_O_7_	192.01939	1.27	3.304	1.37E-08	0.616	0.872	8.67E-07
9	Phenylalanine	C_9_H_11_NO_2_	165.08591	2.10	2.277	3.10E-05	0.784	0.785	0.003
10	Octanoylcarnitine	C_15_H_29_NO_4_	287.21661	5.50	1.003	1.52E-06	0.671	0.801	7.67E-06
11	Glycocholic acid	C_26_H_43_NO_6_	465.30295	6.21	1.333	9.60E-05	2.588	0.779	3.27 E-04
12	Decanoylcarnitine	C_17_H_33_NO_4_	315.24823	6.25	1.033	9.68E-06	0.604	0.812	3.54E-05
13	Cholic acid	C_24_H_40_O_5_	408.28140	6.80	2.060	0.010	4.751	0.779	0.021
14	Glycoursodeoxycholic acid	C_26_H_43_NO_5_	449.30765	6.93	2.325	0.004	1.763	0.726	0.009
15	N-Succinyl-2,6-diaminoheptanedioate	C_11_H_18_N_2_O_7_	289.10617	7.20	1.788	5.92E-12	0.485	0.899	6.01E-11
16	16-HETE	C_20_H_32_O_3_	320.25098	8.03	1.016	8.12E-13	0.393	0.810	7.50E-06
17	LysoPC(16:0)	C_24_H_50_NO_7_P	495.33951	8.68	12.443	1.81E-20	0.565	0.970	1.19E-17
18	LysoPC(18:1)	C_26_H_52_NO_7_P	521.35571	8.87	11.090	4.10E-19	0.663	0.950	6.38E-19
19	9,10-Epoxyoctadecenoic acid	C_18_H_32_O_3_	296.22832	9.22	3.145	0.002	0.660	0.706	0.008
20	Arachidonic acid	C_20_H_32_O_2_	304.23315	10.03	5.301	6.77E-09	0.635	0.845	4.45E-08
21	Oleamide	C_18_H_35_NO	281.27872	10.14	2.071	3.50E-23	0.092	0.502	4.01E-05

OSCC, oral squamous cell carcinoma; HC, healthy control; FC, fold change; RT, retention time; VIP, variable importance in projection; FC, fold change; AUC, area under curve; FDR, false discovery rate, calculation based on Benjamini and Hochberg.

**Table 4:** Metabolites different between OELP and HC

ID	Name	Formula	Molecular	RT	VIP	*P*	FC	AUC	FDR
1	LysoPC(18:1)	C_26_H_52_NO_7_P	521.34786	9.22	1.523	1.87E-17	0.668	0.838	2.94E-16
2	Oleamide	C_18_H_35_NO	281.27872	10.14	2.108	5.63E-25	0.069	0.760	3.18E-23
3	Chiro-inositol	C_6_H_12_O_6_	180.05556	3.25	1.509	0.006	0.908	0.783	0.013037
4	Paraxanthine	C_7_H_8_N_4_O_2_	180.05556	3.25	1.500	8.26E-08	16.416	0.742	2.98E-05
5	Cysteine	C_3_H_7_NO_2_S	121.01138	4.47	1.986	2.38E-37	0.418	0.949	1.11E-08
6	2,4-Dihydroxybenzoic acid	C_7_H_6_O_4_	154.01843	6.34	1.496	0.007	0.243	0.766	0.32
7	Arachidonic acid	C_20_H_32_O_2_	304.23322	10.03	3.621	1.98E-04	1.159	0.808	5.65E-04
8	Uridine	C_9_H_12_N_2_O_6_	244.06238	1.00	1.034	6.86E-11	0.794	0.836	6.89E-04
9	Indolelactic acid	C_11_H_11_NO_3_	205.06627	4.73	1.010	1.14E-05	1.032	0.812	3.77E-04
10	Monobutyl phthalate	C_12_H_14_O_4_	222.08189	6.52	1.216	2.58E-17	0.900	0.736	8.62E-08
11	Malic acid	C_4_H_6_O_5_	134.01326	1.07	1.073	4.76E-22	0.948	0.840	1.01E-20
12	Lactate	C_3_H_6_O_3_	90.02323	1.01	1.060	0.005	1.068	0.682	0.012
13	Glutamate	C_5_H_9_NO_4_	147.04507	1.03	2.550	4.79E-28	0.871	0.893	2.26E-26
14	Histidine	C_6_H_9_N_3_O_2_	155.06137	0.94	2.105	1.81E-13	13.344	0.916	1.20E-12
15	Uric acid	C_5_H_4_N_4_O_3_	168.02016	1.02	5.045	9.81E-06	1.088	0.782	3.26E-05
16	Citric acid	CH_8_O_7_	192.01939	1.27	3.934	9.63E-13	0.863	0.938	5.74E-12
17	N-Succinyl-2,6-diaminoheptanedioate	C_11_H_18_N_2_O_7_	290.10620	7.19	1.609	6.79E-11	0.496	0.843	3.51E-10
18	Taurine	C_2_H_7_NO_3_S	125.00637	1.00	2.011	1.55E-15	0.892	0.907	1.32E-14

OELP, oral erosive lichen planus; HC, healthy control; FC, fold change; RT, retention time; VIP, variable importance in projection; FC, fold change; AUC, area under curve; FDR, false discovery rate, calculation based on Benjamini and Hochberg.
